# Indirect land use changes of biofuel production – a review of modelling efforts and policy developments in the European Union

**DOI:** 10.1186/1754-6834-7-35

**Published:** 2014-03-07

**Authors:** Serina Ahlgren, Lorenzo Di Lucia

**Affiliations:** 1 Department of Energy and Technology, Swedish University of Agricultural Sciences, Uppsala, Sweden; 2 Department of Technology and Society, Lund University, PO Box 118, SE 221 00 Lund, Sweden

**Keywords:** ILUC, review, uncertainty, policy, EU

## Abstract

The issue of indirect land use changes (ILUC) caused by the promotion of transport biofuels has attracted considerable attention in recent years. In this paper, we reviewed the current literature on modelling work to estimate emissions of greenhouse gases (GHG) caused by ILUC of biofuels. We also reviewed the development of ILUC policies in the EU. Our review of past modelling work revealed that most studies employ economic equilibrium modelling and focus on ethanol fuels, especially with maize as feedstock. It also revealed major variation in the results from the models, especially for biodiesel fuels. However, there has been some convergence of results over time, particularly for ethanol from maize, wheat and sugar cane. Our review of EU policy developments showed that the introduction of fuel-specific ILUC factors has been officially suggested by policymakers to deal with the ILUC of biofuels. The values proposed as ILUC factors in the policymaking process in the case of ethanol fuels are generally in line with the results of the latest modelling exercises, in particular for first-generation ethanol fuels from maize and sugar cane, while those for biodiesel fuels are somewhat higher. If the proposed values were introduced into EU policy, no (first-generation) biodiesel fuel would be able to comply with the EU GHG saving requirements. We identified a conflict between the demand from EU policymakers for exact, highly specific values and the capacity of the current models to supply results with that level of precision. We concluded that alternative policy approaches to ILUC factors should be further explored.

## Introduction

The use of bioenergy involves use of land for production of, for example, harvest residues, crops or forestry, so increased demand for bioenergy can cause land use changes (LUC), which can have many implications on the economic, social and environmental sustainability of bioenergy. The LUC directly associated with a bioenergy project are referred to as DLUC, for example, when converting one type of land use to a bioenergy plantation. Indirect LUC (ILUC) are the changes in land use that take place as a consequence of a bioenergy project, but are geographically disconnected to it. For example, displaced food or feed producers may re-establish their operations elsewhere by converting natural ecosystems to agricultural land or, due to macro-economic factors, the losses in food/feed/fibre production caused by the bioenergy project may cause an expansion of the total agricultural area, or an intensification of its use.

Although LUC due to increased demand for bioenergy were first discussed in the 1990s (see for example, [1,2]), the debate on indirect LUC caused by bioenergy production intensified with the publication of two studies in 2008 [3,4]. Both studies demonstrated that ILUC could increase carbon emissions following biofuel expansion to such a level that the life-cycle greenhouse gas (GHG) emissions were higher than with fossil fuels. These studies also had impact on policymaking. Within the EU, in 2009 the Renewable Energy Directive (RED) and Fuel Quality Directive (FQD) were introduced with a set of sustainability criteria for biofuels and bioliquids used to achieve the Directive targets^a^. One of these criteria is that the use of biofuels must ensure at least 35% reduction in GHG emissions compared with fossil fuels^b^. The threshold will increase to 50% after 2017 (60% for installations that enter operation after 2017). However, the methodology for calculating GHG emissions contained in the Directives does not account for emissions due to ILUC. Only in October 2012 did the EC put forward a proposal to amend the Directives and address GHG emissions generated through ILUC.

One of the major problems with including ILUC in policy is the uncertainty related to the quantification of the GHG emissions [5]. Quantification of GHG emissions due to ILUC is very different from quantification of direct changes, as the theory in ILUC modelling is based on economic market reactions to increasing demand for biofuels, whereas quantifying direct changes relies more on natural science. ILUC are not observable. A wheat-growing farmer in Europe will not see any indirect effects of his or her actions, and it can never be proven that a certain land use in Brazil, for example, is the effect of the European farmer’s change from producing wheat for food to wheat for ethanol. The links are complex and impossible to attribute to a certain field.

It is common to use economic equilibrium models to estimate ILUC. These tools are complex optimisation models studying the entire global economy or a specific sector, for example, agriculture. Economic models assume that perfect markets exist and that equilibrium is reached when demand equals supply in the economy [6]. There are several different economic models available, which have been developed and used by researchers for many years. Among the most commonly used are, for example, the Global Trade Analysis Project (GTAP) model developed by Purdue University, the Food Agricultural Policy Research Institute and Center for Agricultural and Rural Development (FAPRI-CARD) model developed by FAPRI together with Iowa State University, and the Modeling International Relationships in Applied General Equilibrium (MIRAGE) model developed by the European Commission French National Institute for Agricultural Research (INRA), the UN and the World Trade Organization. Economic models typically assess changes associated with the implementation of a policy, such as promotion of biofuel. The LUC are often calculated as the difference between scenarios with and without implementation of such a policy. Most economic models are complex and require in-depth understanding of the way they are organised [7,8].

Several alternatives to the economic models have been developed using different approaches. The feature these other methods have in common is that they try to use descriptive methods rather than complex optimisation models. For example, one such model allows reference expert groups to describe likely scenarios of market reaction to increased demand for biofuel [9]. Others use statistics on past LUC to predict future LUC (for example, [10,11]).

The uncertainty in ILUC estimation has been discussed in several papers (for example, [12-14]). There are many explanations for the large variation in modelling results, for example, differences in input data and assumptions. Decisive for the results is the type of land assumed to be affected and the GHG emissions attributed to the LUC, as well as how the emissions are treated over time. LUC, for example, a shift from forest or permanent pasture to annual crops, can lead to large initial carbon losses. However, since the land will continue to produce crops for several years, the carbon losses must be allocated over time, or treated as emission impulses to the atmosphere [8,15,16]. The uncertainty in model results is a problem for policymakers, since it affects the validity of arguments for and against policy support for biofuels. Although the aim of this paper is not to go into details of uncertainty due to underlying assumptions in the models, we return to this in the discussion.

The aim of this paper was to review the recent literature (2009 onwards) reporting efforts to model biofuel ILUC. In our analysis, we evaluated whether results from different types of modelling exercises are comparable and whether there is evidence of result convergence over time. In addition, we reviewed the development of ILUC policies in the EU and compared modelling results with the ILUC values proposed by EU policymakers. We then assessed the possibility of different types of biofuels to comply with the GHG-saving requirements established in the RED and FQD. The paper is intended to give a comprehensive state-of-the-art update of modelling results and policy efforts in the EU, which can be useful to inform future research as well as policy discussions on the topic.

### Methodology for literature review of ILUC modelling

The literature review was based on a previous summary of studies [17], complemented with a search in Google Scholar in October 2013 using the keywords biofuel, ILUC, model and “g CO_2_” for the publication period 2011 to 2013. This yielded 86 hits, of which nine were studies describing models explicitly estimating GHG emissions due to ILUC which were not covered in the previous summary [17].

Most models calculate ILUC on a hectare base, then attribute a GHG emissions factor for LUC and finally allocate the emissions over a number of years and per unit energy of fuel. The allocation of LUC emissions varied in the reviewed literature between 20 and 30 years, most US studies apply 30 years whereas European studies most commonly apply 20 years. In this study, the ILUC data found in the publications were recalculated to a 20-year allocation base in order to be able to compare model results with the ILUC-factors suggested by EU policy-makers, which are allocated over 20 years. Model results are expressed as g CO_2_-eq per MJ biofuel. The studies reviewed were divided into two categories: economic (E) and miscellaneous (M) models. The latter including all non-economic modelling, as mentioned in the introduction. The results were analysed qualitatively, that is, no other processing of the data was done except for the time adjustment.

## Review

### Review of ILUC modelling results

**Figure 1:**
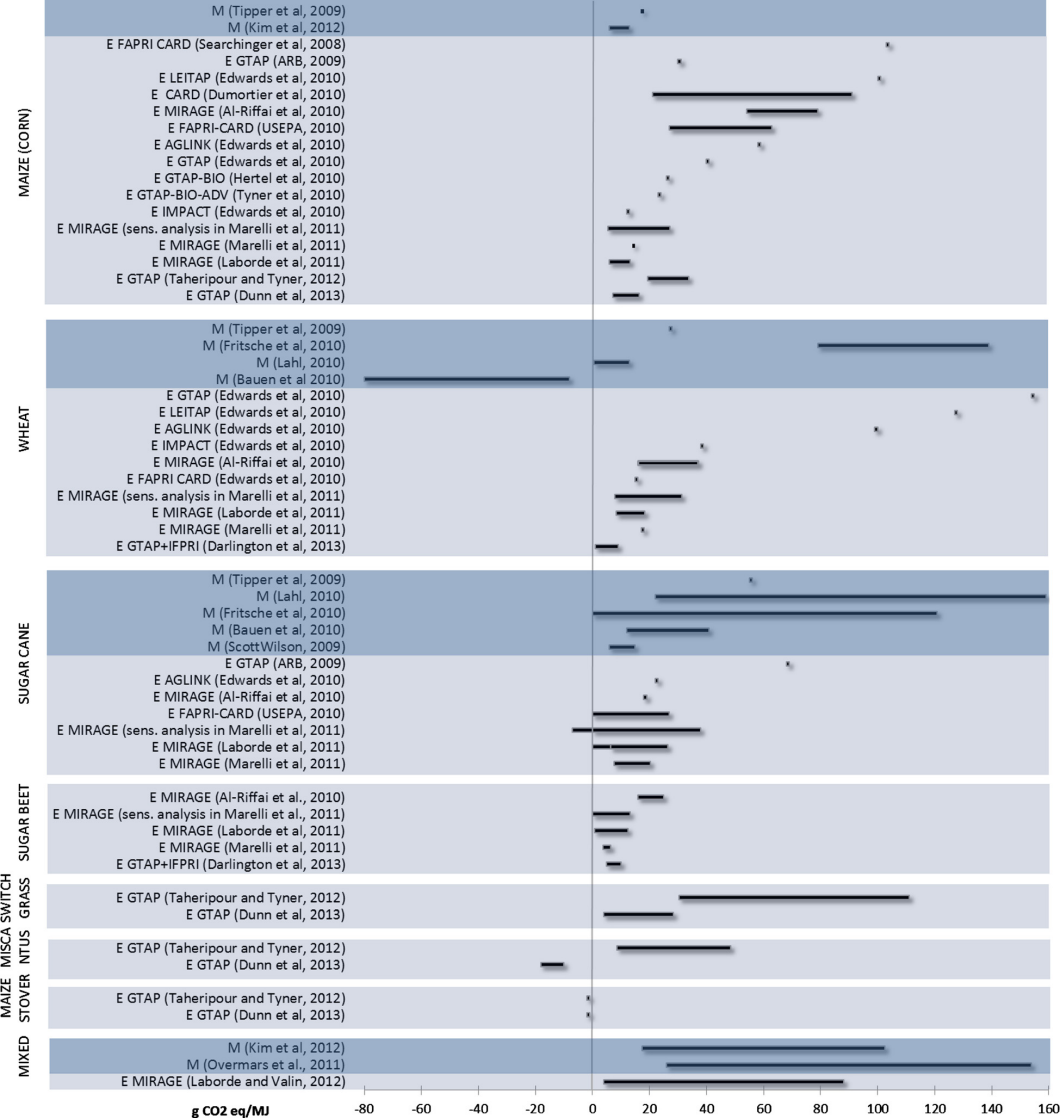
**Review of modelled greenhouse gas (GHG) emissions due to indirect land use change (ILUC) of ethanol biofuels.** All values were recalculated to a 20-year allocation base and are expressed as g CO_2_-eq per MJ ethanol. Some studies show results as intervals (illustrated with lines), others as specific values (illustrated with dots). The studies were divided into two categories; economic modelling (E) and miscellaneous (other) modelling (M). FAPRI-CARD, Food Agricultural Policy Research Institute and Center for Agricultural and Rural Development; GTAP, Global Trade Analysis Project; MIRAGE, Modeling International Relationships in Applied General Equilibrium; IFPRI, International Food Policy Research Institute; LEITAP, the abbreviation indicates the extension of the GTAP model developed at the LEI (Landbouw Economisch Instituut) in The Hague; AGLINK, Worldwide Agribusiness Linkage Program.

**Figure 2:**
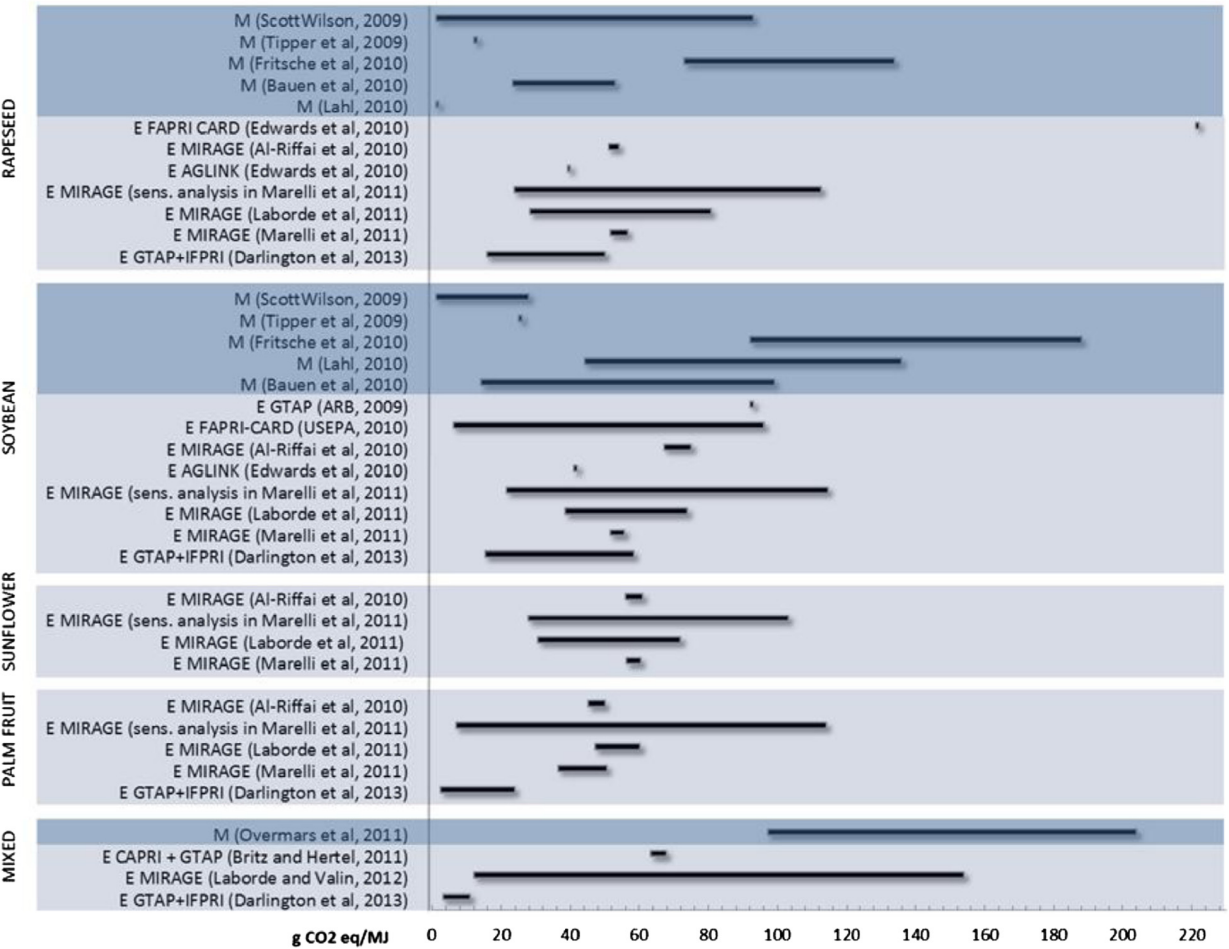
**Review of modelled greenhouse gas (GHG) emissions due to indirect land use change (ILUC) of biodiesel.** All values were recalculated to a 20-year allocation base and are expressed as g CO_2_-eq per MJ biodiesel. Some studies show results as intervals (illustrated with lines), others as specific values (illustrated with dots). The studies were divided into two categories; economic modelling (E) and miscellaneous (other) modelling (M). FAPRI-CARD, Food Agricultural Policy Research Institute and Center for Agricultural and Rural Development; GTAP, Global Trade Analysis Project; MIRAGE, Modeling International Relationships in Applied General Equilibrium; AGLINK, Worldwide Agribusiness Linkage Program. Note the different scale of the x-axis compared with Figure 1.

The results of the literature review are presented in Figure 1 for ethanol fuels and Figure 2 for biodiesel. The results expressed in g CO_2_-eq per MJ fuel are arranged by date of publication, type of model (economic or miscellaneous) and maximum values. An additional file shows the full list of references for the studies (Additional file 1).

The review showed that within the selected sample of papers, most modelling was carried out for ethanol, especially with maize as feedstock; that most studies employed economic equilibrium models,and that the majority of the studies were published in 2010 (9 out of 22 studies).

The variation in the results was found to be large. Some of the studies plotted the results as a single number, while others presented a range based on the statistical distribution given by the authors or the sensitivity analysis performed in the studies. This meant that it was not possible to calculate any average values from Figures 1 and 2. Some feedstock displayed more convergence in the results, especially in the results from the economic models. For example, the economic models for sugar cane ethanol yielded ILUC values of between −5 and 25 g CO_2_-eq/MJ (excluding one value of 69 g CO_2_-eq/MJ in the California Low Carbon Fuel Standard from 2009 [18], which is under revision), whereas the miscellaneous model produced values of between −1 and 159 g CO_2_-eq/MJ. However, this does not necessarily mean that the economic models are less uncertain. An alternative explanation could be that economic models do not reflect the full uncertainty associated with quantification of ILUC, as comprehensive sensitivity analysis is often lacking [13], and many of the reviewed alternative models test results using extreme values regarding, for example, carbon stock in soil. Further, as previously pointed out [19], economic models fail to include other important ILUC drivers such as political, cultural, demographic and environmental issues. On the other hand, many of the alternative models risk missing dynamic and interlinked ILUC driving-factors.

Maize stover and sugar beet were identified as feedstocks with low emissions in all studies. We did not analyse the reason for this, but as maize stover is a by-product from maize production, it is not unreasonable for it to have a low ILUC effect. For sugar beet, the high yields obtained with this crop can be one explanation for the low emissions, as higher yields imply lower land use per MJ of produced biofuel and, therefore, less risk of displacement of other agricultural activities (more detailed analysis is needed to explain low ILUC for sugar beet).

The largest variation in results was seen for wheat ethanol and soybean biodiesel. However, over time there was some convergence of results, particularly regarding ethanol from maize, which has undergone much modelling effort. Sugar cane and wheat showed similar patterns. The values reported for biodiesel fuels showed greater variation than those for ethanol. For biodiesel, a few studies reported ILUC values lower than 10 g CO_2_-eq/MJ, whereas for ethanol several studies showed a range of emissions in which the lowest values were below 10 g CO_2_-eq/MJ or even close to, or below, zero in some cases.

The review also revealed that only a handful of studies to date have analysed advanced biofuels. Both [20] and [21] modelled switchgrass, miscanthus and maize stover for ethanol production, but reached different conclusions, with [20] reporting lower ILUC emissions for both switchgrass and miscanthus to ethanol. We did not analyse the reason for this, but it can be noted that the two studies used different assumptions regarding, for example, the modelled quantity of biofuels and different models to estimate carbon losses due to the LUC.

### EU policy to address ILUC of biofuels

EU policymakers have been struggling for years with the issue of how to deal with the ILUC of biofuels. Already in 2008, during the formulation of the RED and the revision of the FQD, the issue of ILUC took centre stage [22]. According to the final text of the Directives in 2009, the impacts of ILUC had to be investigated further by the EC. In a report released in December 2010, the EC acknowledged that ILUC can reduce the GHG emission-savings associated with biofuels, but it also highlighted the existence of uncertainties and limitations associated with the quantification of indirect emissions in the available models [23]. However, the report did not suggest a concrete approach for tackling the risk of negative ILUC and the related GHG emissions^c^.

Work in the following two years was dedicated to reducing the uncertainties and limitations in the scientific knowledge. The European Commission (EC) launched a number of studies on the ILUC of biofuels^d^, but only in autumn 2012, nearly two years after the date set by the RED and FQD, did the EC present a proposal to address the risk of negative ILUC [24]. The measures contained in the proposal are to: (i) cap the contribution of conventional (food-crop-based) biofuels to 5% of total motor fuel consumption; (ii) bring forward the introduction of the 60% minimum GHG-saving threshold for new installations to 2014; (iii) encourage the diffusion of advanced biofuels by double or quadruple counting their contribution toward the targets of the RED, and (iv) require Member States and fuel suppliers to report the estimated ILUC emissions, employing feedstock-specific ILUC factors (12 g CO_2_-eq/MJ for cereals and other starch-rich crops, 13 g CO_2_-eq/MJ for sugars and 55 g CO_2_-eq/MJ for oil crops) [24]. These ILUC factors are based on the results of the general equilibrium economic model International Food Policy Research Institute (IFPRI)-MIRAGE-BioF [25] and are introduced only for reporting purposes^e^.

The EC proposal is currently being debated within the European Parliament (EP) and Council of the European Union (CEU). The EP adopted a common position on the proposal in September 2013 [26], according to which advanced biofuels should supply at least 2.5% of the energy used for transportation in 2020, and the share of food-crop-based biofuels should be limited to 6% on an energy basis. ILUC factors might be accounted for in the EU sustainability certification system only after 2020. The EC proposal has also been debated within the CEU during 2013. On several occasions the Ministries of Environment and Energy have heavily criticised it, but have failed to produce a common position on the issue. In October 2013, the Irish presidency of the Council advanced a position that calls for a 7% cap on the use of food crops and a minimum share of 2% for advanced biofuels by 2020, but does not mention the introduction of ILUC factors [27]. The negotiation of the proposal will continue throughout 2014, but the chances of adopting a final text before 2015 are now meagre, particularly owing to the parliamentary elections in spring 2014.

### ILUC – public policies and scientific knowledge

**Figure 3:**
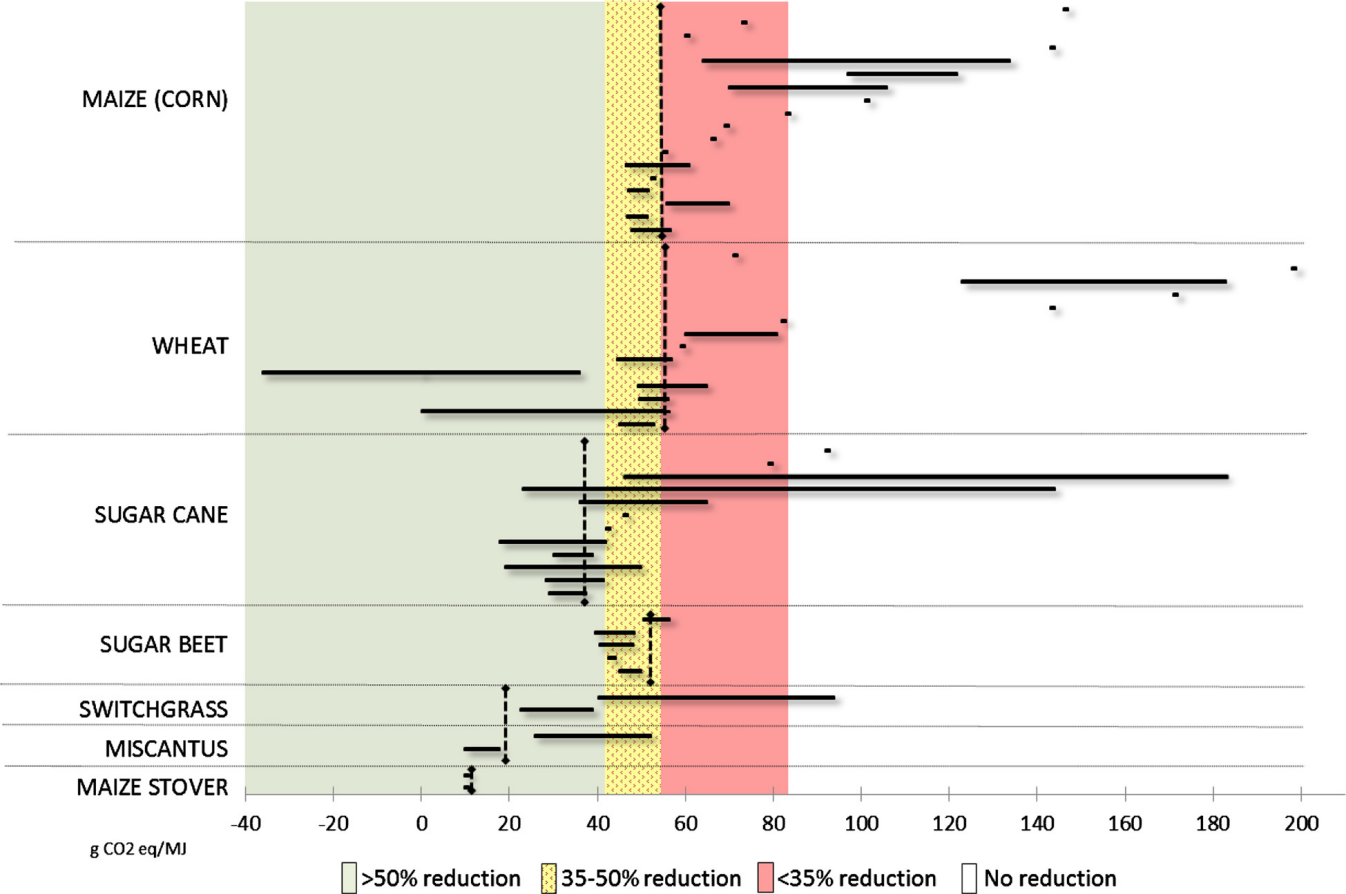
**Estimates of total GHG emissions for ethanol fuels.** Black bars show scientific estimates and include direct emissions as default values established in the Renewable Energy Directive (RED) and Fuel Quality Directive (FQD), and indirect land use change (ILUC) factors from the modelling exercises presented in Figure 1. Dashed black lines show policy estimates and include direct emissions as default values established in the RED and FQD and indirect emissions as estimated in [24].

**Figure 4:**
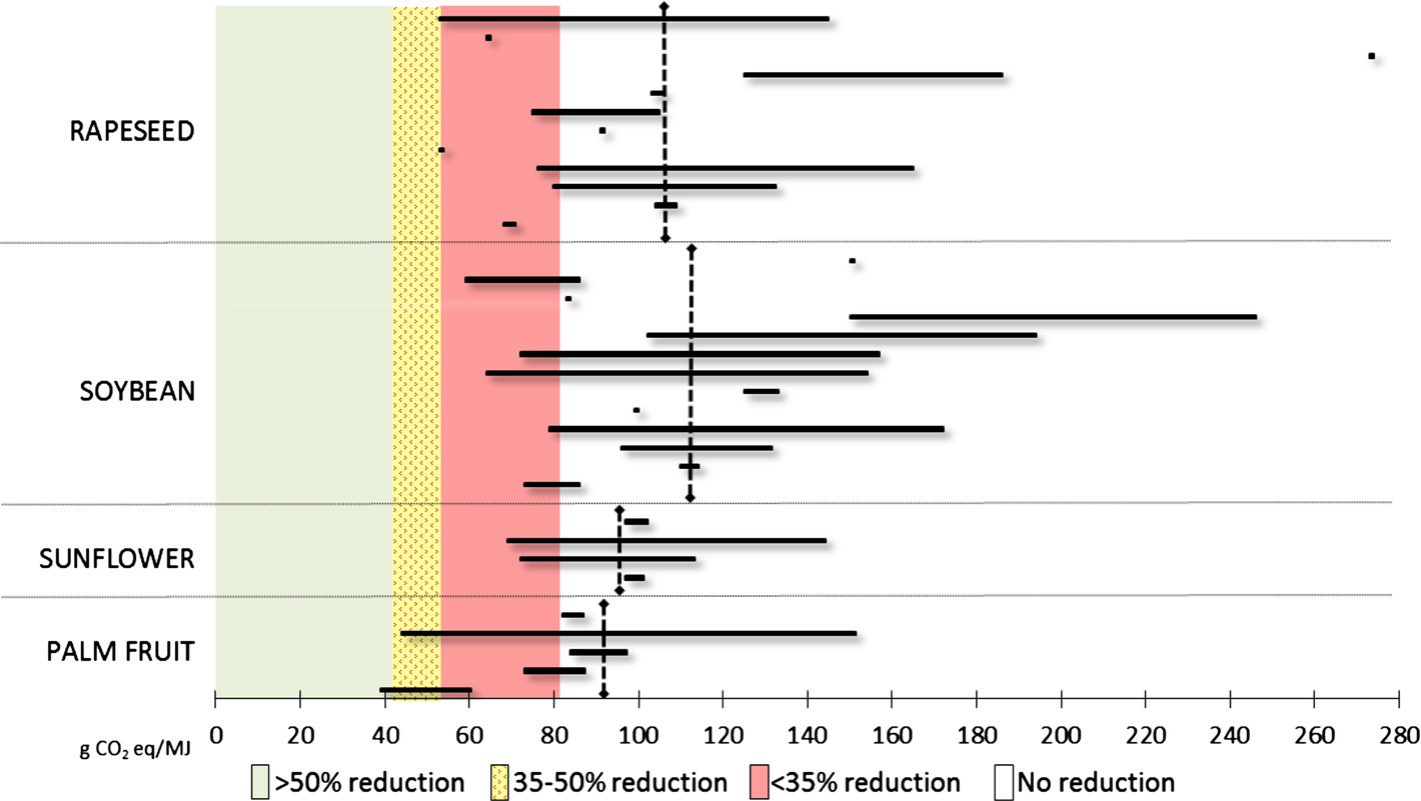
**Estimates of total GHG emissions for biodiesel fuels.** Black bars show scientific estimates and include direct emissions as default values established in the Renewable Energy Directive (RED) and Fuel Quality Directive (FQD), and indirect land use change (ILUC) factors from the modelling exercises presented in Figure 2. Dashed black lines show *policy* estimates and include direct emissions as default values established in the RED and FQD and indirect emissions as estimated in [24].

The recent policy developments in the EU can be viewed in light of the scientific knowledge about ILUC provided by the modelling exercises reviewed above (Review of ILUC modelling results). Policy estimates of total GHG emissions (dashed black lines) are compared with the scientific estimates (black bars) in Figures 3 and 4 to evaluate policymakers’ decisions in light of the available scientific knowledge. Policy-estimated emissions include indirect emissions (ILUC factors) proposed by the EC in 2012 and direct emissions established in the RED and FQD (default values). The results from the modelling studies reviewed are sorted in order of publication date (oldest first) and thereafter in order of maximum value (highest first), but without distinction into model types. Moreover, Figures 3 and 4 show the GHG-saving thresholds established in the RED and FQD. Although these levels represent the political ambitions with biofuels as climate-change mitigation means, they can be evaluated against the levels of total GHG emissions estimated by policymakers and scientists in order to assess the relative performance of ethanol and biodiesel from different feedstock and, more importantly, how the introduction of the policy values will affect each type of biofuel.

In the case of ethanol fuels, it can be seen that the policy values are generally in line with the results of the latest modelling exercises, in particular for first-generation ethanol fuels from maize and sugar cane (Figure 3). However, in the case of advanced ethanol fuels, the fit between policy values and scientific estimates is less evident (except for the case of maize stover)^f^. Compared with the minimum GHG-saving requirements established in the EU Directives, all types of ethanol fuels will be able to comply with the 35% minimum reduction requirement (assuming limited improvements with direct emissions of wheat ethanol), whereas the 50% requirement will be difficult to fulfil for all but sugar cane ethanol and second-generation ethanol fuels.In the case of biodiesel fuels (Figure 4), it can be seen that the policy estimates contained in the EC proposal do not appear to be in line with the modelling results reviewed earlier (Review of ILUC modelling results), or at least not as much as in the case of ethanol fuels. The EC values for biodiesel are generally higher than the range of values reported in the modelling exercises. Even in cases for which we observed some level of convergence of models results, for example, soybean biodiesel, the EC values are considerably higher. If the proposed values were to be introduced into the EU policy to assess compliance with the minimum saving requirements, none of the (first-generation) biodiesel fuels would be able to fulfil the 35%, let alone the 50%, reduction requirement. Policy estimates of total GHG emissions of biodiesel fuels are (substantially) higher than those of fossil fuels, whereas scientific estimates show lower levels, even though the potential for savings of GHG emissions compared with fossil fuels is limited.

## Conclusions

Our review of recent ILUC modelling exercises revealed that most studies employ economic equilibrium modelling and focus on ethanol fuels, especially with maize as feedstock, while only a small number of studies to date have modelled advanced biofuels. It also revealed that in spite of some convergence of results over time, particularly for ethanol from maize, wheat and sugar cane, there is still a major variation in the results from the models, especially for biodiesel fuels.

There are many reasons for the variation in the results found in the review. They can be grouped into three major issues: (i) structural components of the models (ii) input data and assumptions and (iii) treatment of carbon stock changes, both concerning the amount of released or sequestered carbon, and how carbon emissions are treated over time.

i) Structural components of the models: economic models (the most commonly used type of ILUC models) were originally developed for quantitative analysis of global economic and political issues rather than assessing ILUC. They adopt different world views and, therefore, have different assumptions about the development of oil prices, and food prices, et cetera [28]. Economic models such as GTAP and MIRAGE (general equilibrium models) study entire economies, whereas others, such as AGLINK (partial equilibrium models), study only specific sectors. Models differ also in their geographical resolution as some study the system at country level, while others aggregate larger, regional areas. Further, the commodity-level resolution varies among models. Whereas some models, for example, IMPACT, study cereals, others, such as CAPRI, can differentiate between different types of cereals. Finally, other issues such as the possibility to model trade of biofuels or the expansion of agricultural land into different types of land uses (only pasture land, only forest, or both) explains some of the variation in model results. For a more in-depth comparison of these issues among models see previous work as examples [12,28-30].

ii) Input data and assumptions: the studies analyse different policies and use different start and end points in time. They assume policy target will be achieved by different ratios of biodiesel and ethanol and only in a few cases include second generation biofuels. An important set of differences concerns assumptions about harvest levels and raw material use per MJ of biofuel and assumptions about the amount and value of by-products, with some studies (for example, IMPACT and LEITAP models reported in [28]) not accounting for by-products at all. In addition, assumptions about how demand for different commodities depends on commodity price (the so-called elasticity factors) are of great importance and vary significantly among the models. Finally, land prices and costs of land conversion are major assumptions influencing the results of ILUC models [8,12,15,16,30-33].

iii) Treatment of carbon stocks changes (including above and below ground biomass): assumptions regarding the type of land that will be converted and the related emissions are important for the variation in results of ILUC models [12]. As these assumptions are usually not included in economic models, additional models need to be added. For example, very high ILUC factors of biodiesel can in some instances be explained by assumptions about ILUC on peat soils in South East Asia [12], see for example, previously reported studies [34].

Our review of the development of EU policies to address the GHG emissions associated with ILUC for production of biofuels showed that EU policymaking suffers from a number of inconsistencies and weaknesses. The approaches officially suggested by policymakers in recent years recognise, to different degrees, the necessity of accounting for ILUC in biofuel support policies. The ILUC factors selected by policymakers for this purpose are very specific in terms of g CO_2_-eq per MJ of biofuel, which is clearly at odds with the uncertainty in results emerging from modelling exercises to date. Thus, there is a conflict between the demand from EU policymakers for exact, highly specific values and the capacity of the current models to supply results with that level of precision.

The uncertainty of ILUC estimates may be reduced in the future as better models and better data reduce the epistemological uncertainty associated with lack of knowledge of system behaviour [35]. We observed in our review some convergence of results over time, particularly regarding ethanol from maize (which has undergone much modelling effort), sugar cane and wheat. However, uncertainty will not be eliminated. The modelling results produced to date indicate that due to the complexity of the global economy, no significant reduction in model uncertainty should be expected in the near future [36]. Furthermore, predictions of future changes in complex natural and socio-technical systems are intrinsically uncertain. Future ILUC will be dependent not only on economic reactions, but also on other (unforeseen) factors such as agricultural and trade policies in different parts of the world. This so-called variability uncertainty [35] is not reducible and results in a variety of valid scientific standpoints.

The gap between demand by policymakers for indisputable evidence and final answers and the lack of conclusiveness and definitiveness in the knowledge generated by scientific models is visually displayed in Figures 3 and 4. This gap fuels the abundant criticisms linked to the introduction of ILUC factors in EU policies. Considering that the effectiveness of the introduction of (controversial) ILUC factors on the LUC of biofuel policies has not been modelled, and is probably outside the reach of current models, we must conclude that alternative policy approaches should be further explored. One such alternative approach would be to place a cap on food-crop-based biofuels, as proposed by the EC in 2012 [24]. However, the cap suggested by the EC is a very general measure, which does not reflect the real risk of ILUC of different types of biofuels. It aims at regulating the indirect effects of biofuel promotion without being able to measure them. Hence, it is not an effective measure to reduce the risk of negative LUC. We believe that an overall strategy for land use is more urgently needed than ILUC factors or a cap on food crop-based biofuels.

## Endnotes

^a^The RED sets a binding target of 10% renewable fuels, including biofuels, in transportation by 2020 for each Member State, while the FQD mandates fuel suppliers to lower the GHG emissions by 6% for each unit of energy from fuel sold by 2020.

^b^The fossil fuel comparator for calculation of GHG reductions is at present 83.8 g CO_2_-eq/MJ, but is under revision.

^c^The report identified four options: (i) take no action for the time being, while continuing to monitor; (ii) increase minimum GHG saving thresholds; (iii) introduce additional sustainability requirements on certain categories of biofuels; and (iv) attribute a quantity of GHG emissions to biofuels reflecting the estimated ILUC impact. These options have been evaluated in [25] and [5].

^d^These are available on the EC website (http://ec.europa.eu/energy/renewables/studies/land_use_change_en.htm).

^e^In a leaked draft version of the EC proposal, ILUC factors were included in the methodology to calculate the GHG balance of biofuels for compliance with the minimum GHG saving requirements.

^f^Note that the EC proposal assigns an ILUC factor of zero to all advanced biofuels.

## Abbreviations

CEU: Council of the European Union; CO_2_-eq: carbon dioxide equivalents; DLUC: direct land use change; EC: European commission; EP: European parliament; EU: European Union; FAPRI-CARD: Food agricultural policy research institute and center for agricultural and rural development; FQD: Fuel quality directive; GHG: greenhouse gas; GTAP: Global trade analysis project; IFPRI: International food policy research institute; ILUC: Indirect land use change; INRA: French national institute for agricultural research LUC: Land use change; MIRAGE: Modeling international relationships in applied general equilibrium; RED: Renewable energy directive.

## Competing interests

The authors declare that they have no competing interests.

## Authors’ contributions

SA initiated the study and performed the literature review. LDL created the diagrams and performed the policy analysis. Both authors substantially contributed to the analysis and the drafting of the manuscript. All authors read and approved the final manuscript.

## Authors’ information

Serina Ahlgren holds a PhD in agronomy and is currently working as a researcher at the Swedish University of Agricultural Sciences, mainly with life cycle assessment studies of bioenergy systems. Lorenzo Di Lucia is a post-doctoral fellow at Lund University, where he is working on the governance of biofuel systems in the EU and developing countries.

## Supplementary Material

Additional file 1 **List of references from the literature review.** Studies used for the review of indirect land use change (ILUC) models. Click here for file
