# Erratum to: Indirect land use changes of biofuel production – a review of modelling efforts and policy developments in the European Union

**DOI:** 10.1186/s13068-016-0459-4

**Published:** 2016-02-19

**Authors:** Serina Ahlgren, Lorenzo Di Lucia

**Affiliations:** Department of Energy and Technology, Swedish University of Agricultural Sciences, Uppsala, Sweden; Department of Technology and Society, Lund University, Lund, Sweden

## Erratum to: Biotechnology for Biofuels 2014, 7:35 DOI 10.1186/1754-6834-7-35

Following publication of the original article [1] in *Biotechnology for Biofuels*, we have discovered two errors in our study concerning the interpretation of the results of one of the studies we reviewed, the E GTAP + IFPRI study [2]. First, the values we used in Fig. 2 for Rapeseed, Soybean, Palm fruit and Mixed are not correct. Correct values are 2-19, 16-28, 15-24, 3-11 g CO_2_-eq per MJ, respectively. Second, in dialog with one of the authors of the named study, Darlington [3], we have also come to the conclusion that the range of ILUC values in Darlington et al. [2] should be based on Table 17 of their report in which idle/fallow land is included.

Darlington in an email writes: “Table 17 shows the main analysis. Table 14 just shows the results if the analysis is done similarly to the IFPRI analysis, but we point out in the report that we believe idle land should be included in any analysis of LUC values in the EU, and that is included in Table 17”.

Following this, the ILUC values for Darlington et al. [2] used in our study in both Figs. 1 and 2 should read:

**Table:** 

Biofuel	Feedstock	g CO_2_-eq per MJ
Biodiesel	Palm fruit	15-24
	Rapeseed	2-5
	Soybean	16-16
	Mixed	3-3
Ethanol	Wheat	1-3
	Sugar beet	5-7

Darlington points out that the quite low ILUC values compared to all the other numbers in the review are because they included the conversion of idle/fallow land to crops in the EU27 in the analysis, where none of the other analyses have attempted to do that. Thus, it shows the importance of getting the land cover correct before doing LUC modeling.
