# Supplementary material for: Indirect land use changes of biofuel production – a review of modelling efforts and policy developments in the European Union
Source: Biotechnol Biofuels. 2014 Mar 7;7:35. doi: 10.1186/1754-6834-7-35 (PMC4015842; doi:10.1186/1754-6834-7-35)
Supplement: Additional file 1 — List of references from the literature review. Studies used for the review of indirect land use change (ILUC) models. [file 1754-6834-7-35-S1.docx]

**Additional file 1**

List of references from the literature review:

Al-Riffai P, Dimaranan B, Laborde D: **Global Trade and Environmental Impact Study of the EU Biofuels Mandate. Final Report March 2010**. ATLASS Consortium Study by IFPRI for the Directorate General for Trade of the European Commission; 2010

ARB: **Final Regulation Order. Subchapter 10. Climate Change Article 4. Regulations to Achieve Greenhouse Gas Emission Reductions Subarticle 7. Low Carbon Fuel Standard. California Air Resources Board**; 2009

Bauen A, Chudziak C, Vad K, Watson P: **A causal descriptive approach to modelling the GHG emissions associated with the indirect land use impacts of biofuels. Final report. A study for the UK Department for Transport, E4tech**; 2010

Britz W, Hertel TW: **Impacts of EU biofuels directives on global markets and EU environmental quality: An integrated PE, global CGE analysis.** *Agriculture, Ecosystems & Environment* 2011, **142:**102-109

Darlington T, Kahlbaum D, O’Connor D, Mueller S: **Land Use Change Greenhouse Gas Emissions of European Biofuel Policies Utilizing the Global Trade Analysis Project (GTAP) Model. Report by Air Improvement Resource, Inc., (S&T)2 Consultants Inc. and University of Illinois, Chicago**; 2013

Dumortier J, Hayes DJ, Carriquiry M, Dong F, Du X, Elobeid A, Fabiosa JF, Tokgoz S: **Sensitivity of Carbon Emission Estimates from Indirect Land-Use Change. Working Paper 09-WP 493, July 2009.** Center for Agricultural and Rural Development, Iowa State University; 2010

Dunn JB, Mueller S, Kwon H-y, Wang MQ: **Land-use change and greenhouse gas emissions from corn and cellulosic ethanol.** *Biotechnology for biofuels* 2013, **6:**51

Edwards R, Mulligan D, Marelli L: **Indirect Land Use Change from increased biofuels demand. Comparison of models and results for marginal biofuels production from different feedstocks**. JRC Scientific and Technical Reports EUR 24485 EN; 2010

Fritsche UR, Hennenberg K, Hünecke K: **Sustainability Standards for internationally traded Biomass. The “iLUC Factor” as a Means to Hedge Risks of GHG Emissions from Indirect Land Use Change - Working Paper.** Energy & Climate Division, Öko-Institut, Darmstadt Office; 2010

Hertel TW, Golub AA, Jones AD, O'Hare M, Plevin RJ, Kammen DM: **Effects of US maize ethanol on global land use and greenhouse gas emissions: estimating market-mediated responses.** *BioScience* 2010, **60:**223-231

Kim S, Dale BE, Ong RG: **An alternative approach to indirect land use change: Allocating greenhouse gas effects among different uses of land**. *Biomass and Bioenergy* 2012, **46**:447-452

Laborde D, Valin H: **Modelling land-use changes in a global CGE: assessing the EU biofuel mandates with the Mirage-biof model.** *Climate Change Economics* 2012, **3**

Laborde D: **Assessing the land use change consequences of European biofuel policies. Final Report**. ATLASS Consortium Specific Contract No SI2 580403, implementing Framework Contract No TRADE/07/A2 International Food Policy Research Institute, Washington DC. 2011

Lahl U: **An analysis of iLUC and biofuels regional quantification of climate relevant land use change and options for combating it**. BZL Kommunikation und Projektsteuerung GmbH; 2010

Marelli L, Ramos F, Hiederer R, Koeble R: **Estimate of GHG emissions from global land use change scenarios.** Joint Research Centre, European Commission JRC 64430, EUR 24817 EN, DOI 102788/20453; 2011

Overmars KP, Stehfest E, Ros JPM, Prins AG: **Indirect land use change emissions related to EU biofuel consumption: an analysis based on historical data.** *Environmental Science & Policy* 2011, **14:**248-257

Wilson S: **Developing a spreadsheet model for the calculation of the emissions from indirect land use change (ILUC) as a result of biofuel production-explanatory note**; Friends of the Earth**,** 2009. [http://np-net.pbworks.com/w/file/fetch/31633701/FOE%2C%20Scott%20Wilson%28%202009%29%20Dveloping%20a%20speradsheet%20model.%20Biofuels%20research%20explanatory%20note%281%29.pdf]

Searchinger T, Heimlich R, Houghton RA, Dong F, Elobeid A, Fabiosa J, Tokgoz S, Hayes D, Yu T-H: **Use of U.S. croplands for biofuels increases greenhouse gases through emissions from land-use change.** *Science* 2008, **319:**1238-1240

Taheripour F, Tyner WE: **Induced land use emissions due to first and second generation biofuels and uncertainty in land use emissions factors.** In *2012 Annual Meeting, August 12-14, 2012, Seattle, Washington*. Agricultural and applied economics association; 2012

Tipper R, Hutchison C, Brander M: **A practical approach for policies to address GHG emissions from indirect land use change associated with biofuels**. Ecometrica and Greenergy technical paper - TP-080212-A; 2009

Tyner WE, Taheripour F, Zhuang Q, Birur D, Baldos U: **Land use changes and consequent CO2 emissions due to US corn ethanol production: A comprehensive analysis.** Department of agricultural economics, Purdue University; 2010

USEPA: **Renewable fuel standard program (RFS2): Regulatory impact analysis. Assessment and standards division office of transportation and air quality U.S. Environmental Protection Agency. EPA-420-R-10-006**; 2010
